# Supplementary material for: Photosynthate distribution determines spatial patterns in the rhizosphere microbiota of the maize root system
Source: Nat Commun. 2025 Aug 7;16:7286. doi: 10.1038/s41467-025-62550-y (PMC12331955; doi:10.1038/s41467-025-62550-y)
Supplement: Supplementary file 1 — Supplementary Information [file 41467_2025_62550_MOESM1_ESM.pdf]

SUPPLEMENTARY MATERIAL

Photosynthate distribution determines spatial patterns in the rhizosphere microbiota of the maize root system

Sina R. Schultes, Lioba Rüger, Daniela Niedeggen, Jule Freudenthal, Katharina Frindte, Maximilian F. Becker, Ralf Metzner, Daniel Pflugfelder, Antonia Chlubek, Carsten Hinz, Dagmar van Dusschoten, Sara L. Bauke, Michael Bonkowski, Michelle Watt, Robert Koller, Claudia Knief

Supplementary figures

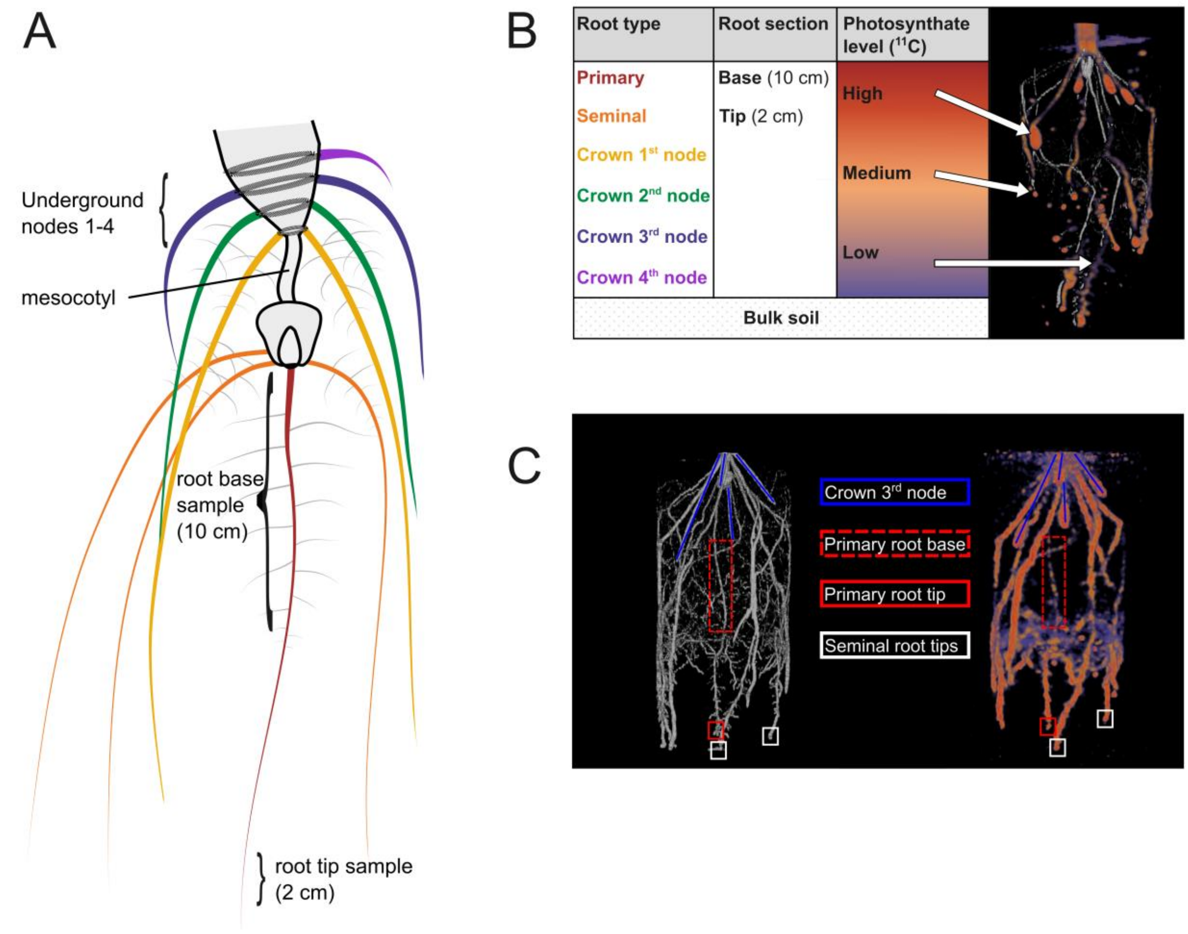

Figure S1 Sampling concept in both studies. **a** Conceptual drawing of a maize root system on harvesting day. The seed-borne root system comprises the primary root and several seminal roots. The shoot-borne root system comprises crown roots originating from consecutive belowground nodes. The internodes between the first four underground nodes usually do not elongate, resulting in a 'triangle' of stacked nodes. The sampled root sections tip and base are illustrated exemplarily on the primary root. **b** The sampling scheme: Root tips and bases of all root types including crown roots from consecutive nodes were collected. Photosynthate levels were defined based on PET/MRI scans. **c** Example of PET/MRI scans and ^{11}C signal intensity categorization using a plant from study II at week 3 after sowing. Clearly visible ^{11}C accumulations at tips of crown roots from the 3rd node (blue) are categorized as root tips with "high" signal intensity. At the root tips of primary, seminal and older crown roots, smaller accumulations were seen and classified as areas with "medium" signal intensity. Root bases, which mostly pass ^{11}C labeled photosynthates without accumulating (e.g. the primary root base, red dashed) were classified as "low" signal intensity areas. Thus, we distinguished between root tips with high ^{11}C signal intensity, root tips with medium signal intensity and root basal regions with low signal intensity.

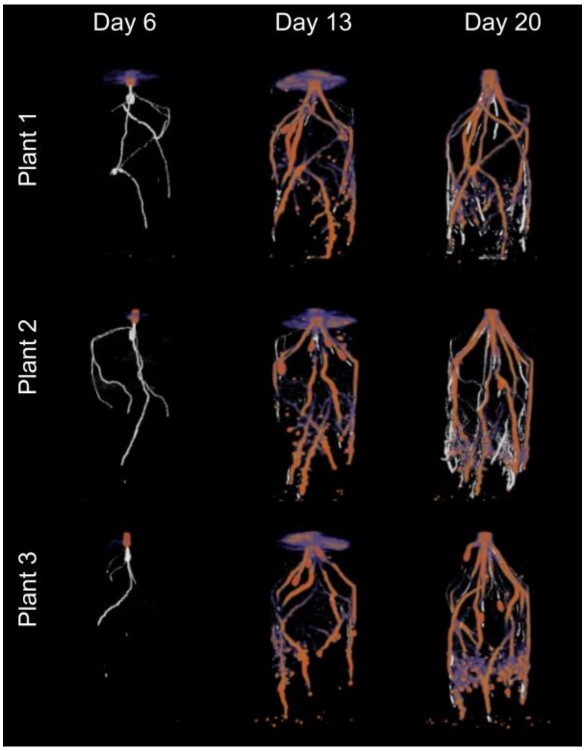

Figure S2 The distribution of fresh, ^{11}C-labelled photosynthates in all plants of study I on day 6, day 13 and day 20 after sowing. Co-registrations of PET scans (colored) and MRI scans (grey) of the same maize plant for images taken at 85-90 minutes after the start of labelling.

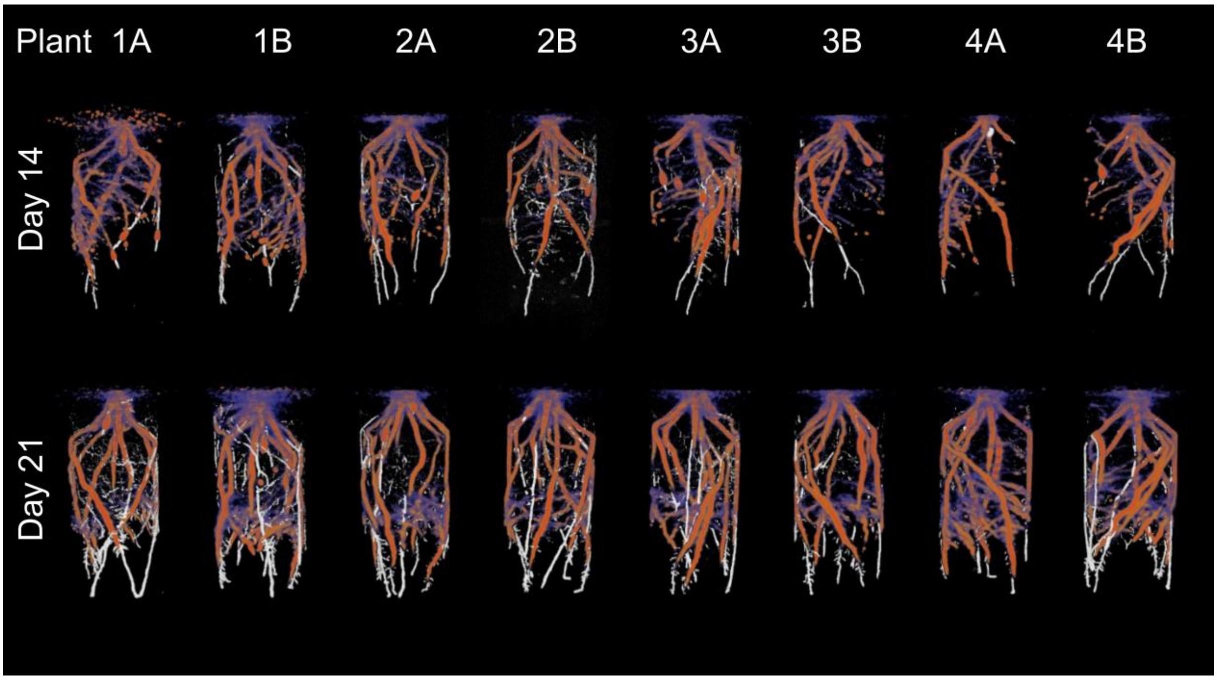

Figure S3 The distribution of fresh, ^{11}C-labelled photosynthates in plants of study II on day 14 and day 21 after sowing. Co-registrations of PET scans (colored) and MRI scans (grey) of the same maize plant for images taken at 85-90 minutes after the start of labelling.

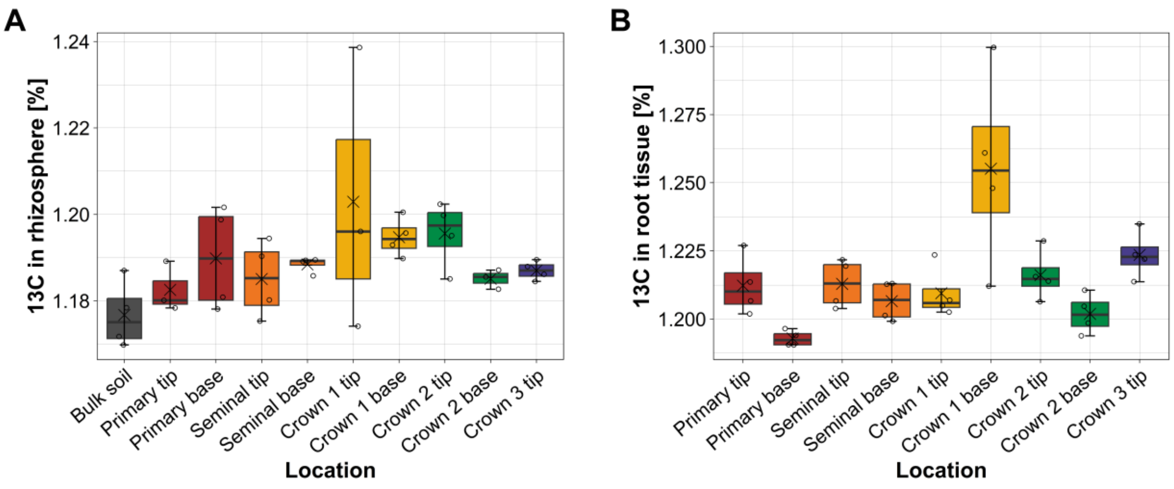

Figure S4 Mass fraction of ^{13}C [%] in the rhizosphere (**a**) and root tissue (**b**) of unlabeled control plants as measured by EA-IRMS (*n* = 4). Boxes span from the first to the third quartiles, the line inside each box represents the median; x represents the mean and the whiskers extend to the last data point within 1.5 times the inter quartile range. Data points outside of whiskers represent outliers. Source data are provided as a Source Data file.

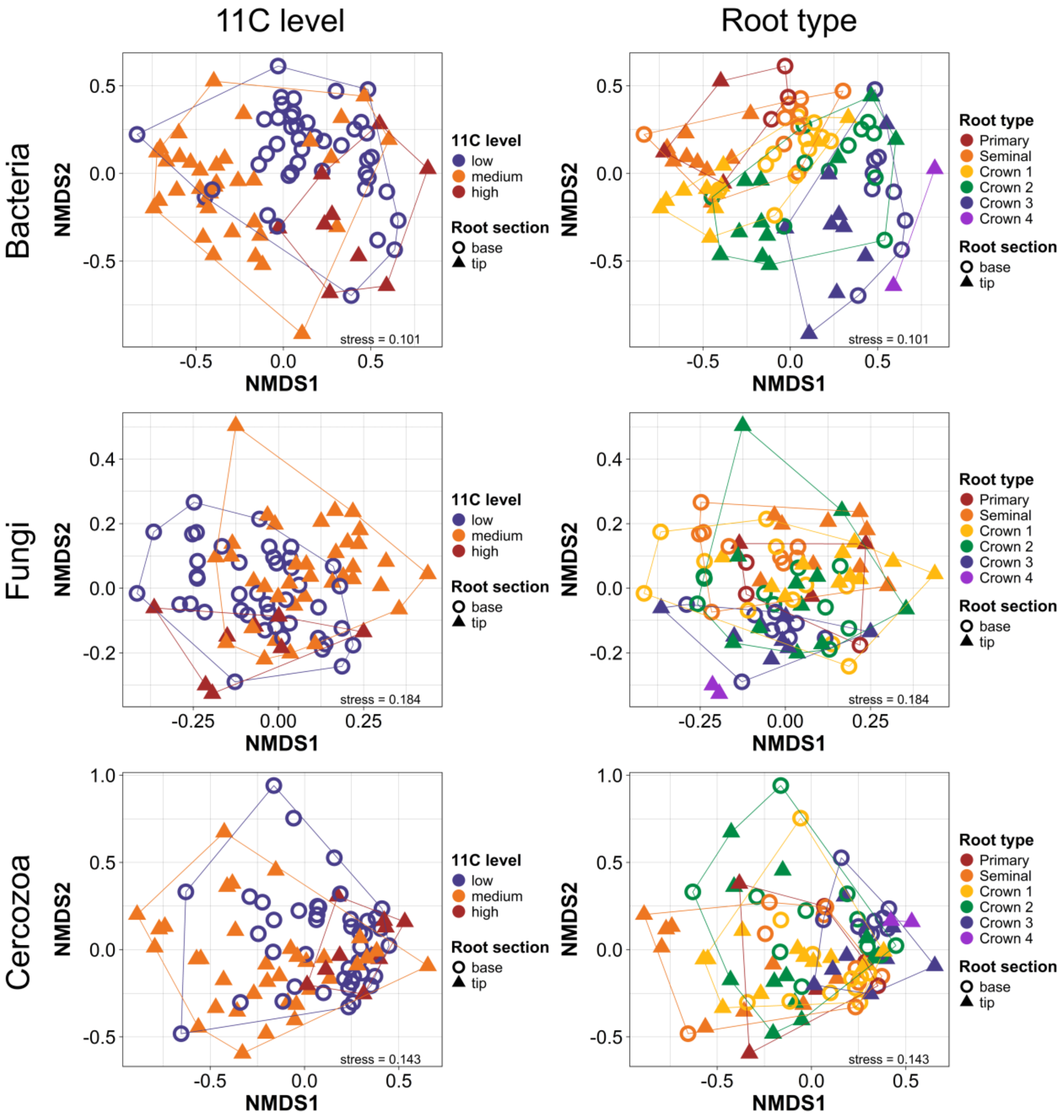

Figure S5 Community composition of the maize rhizosphere microbiota (prokaryotes, fungi, Cercozoa) in study I, with sample coding in dependence on the factors root type, root section and categorical photosynthate levels as identified by ^{11}C-PET. NMDS plots were created using k = 3 dimensions. Source Data are provided as a Source Data file.

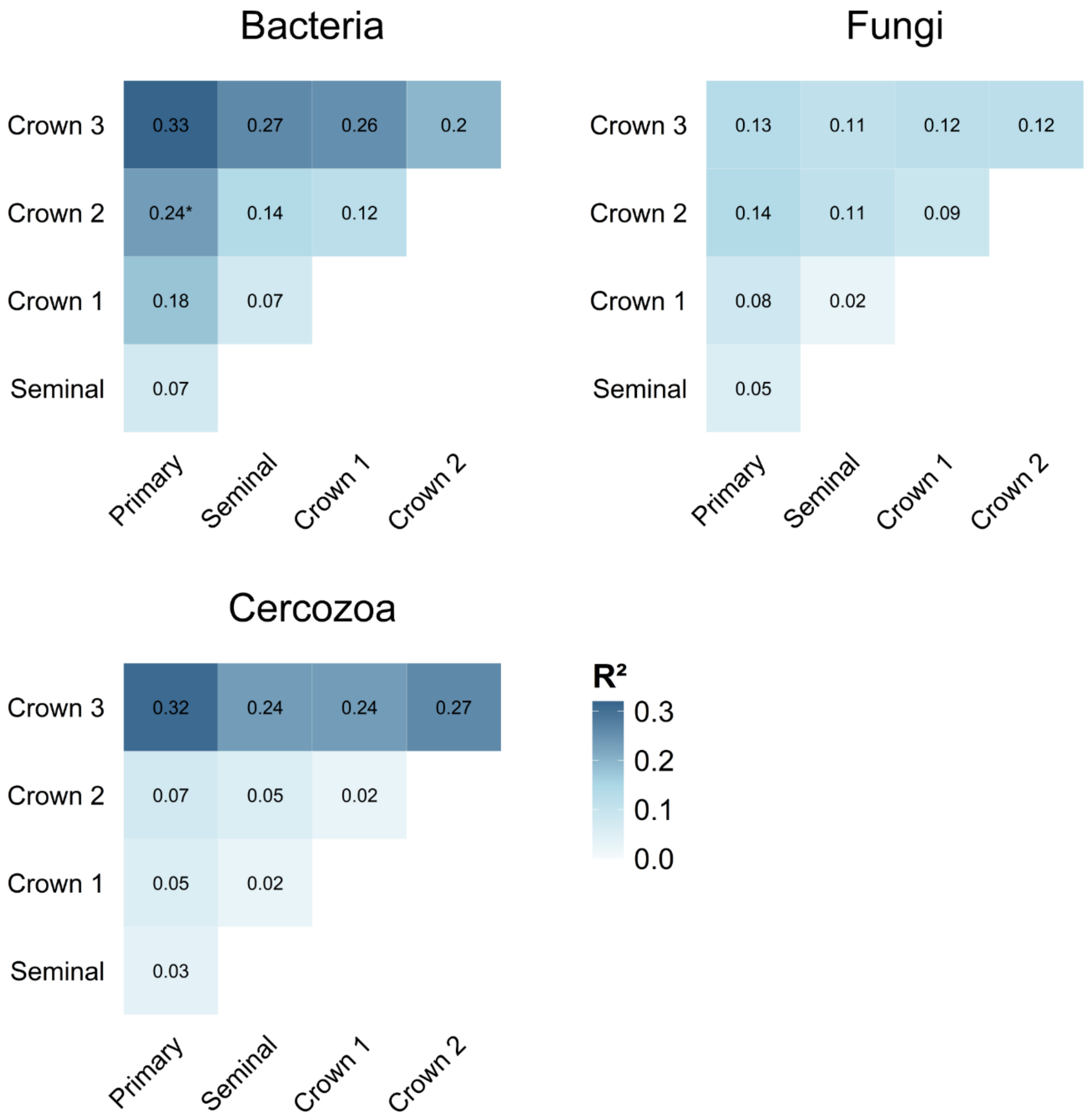

Fig. S6 Heatmaps illustrating results for pairwise PERMANOVA applied to compare microbial community composition between different root types in the ^{13}C-heavy fraction of study II. R^2-values are reported and additionally visualized by color scale, significant differences are indicated by asterisks with * reporting significances upon Bonferroni correction with $p < 0.05$. Source data are provided as a Source Data file.

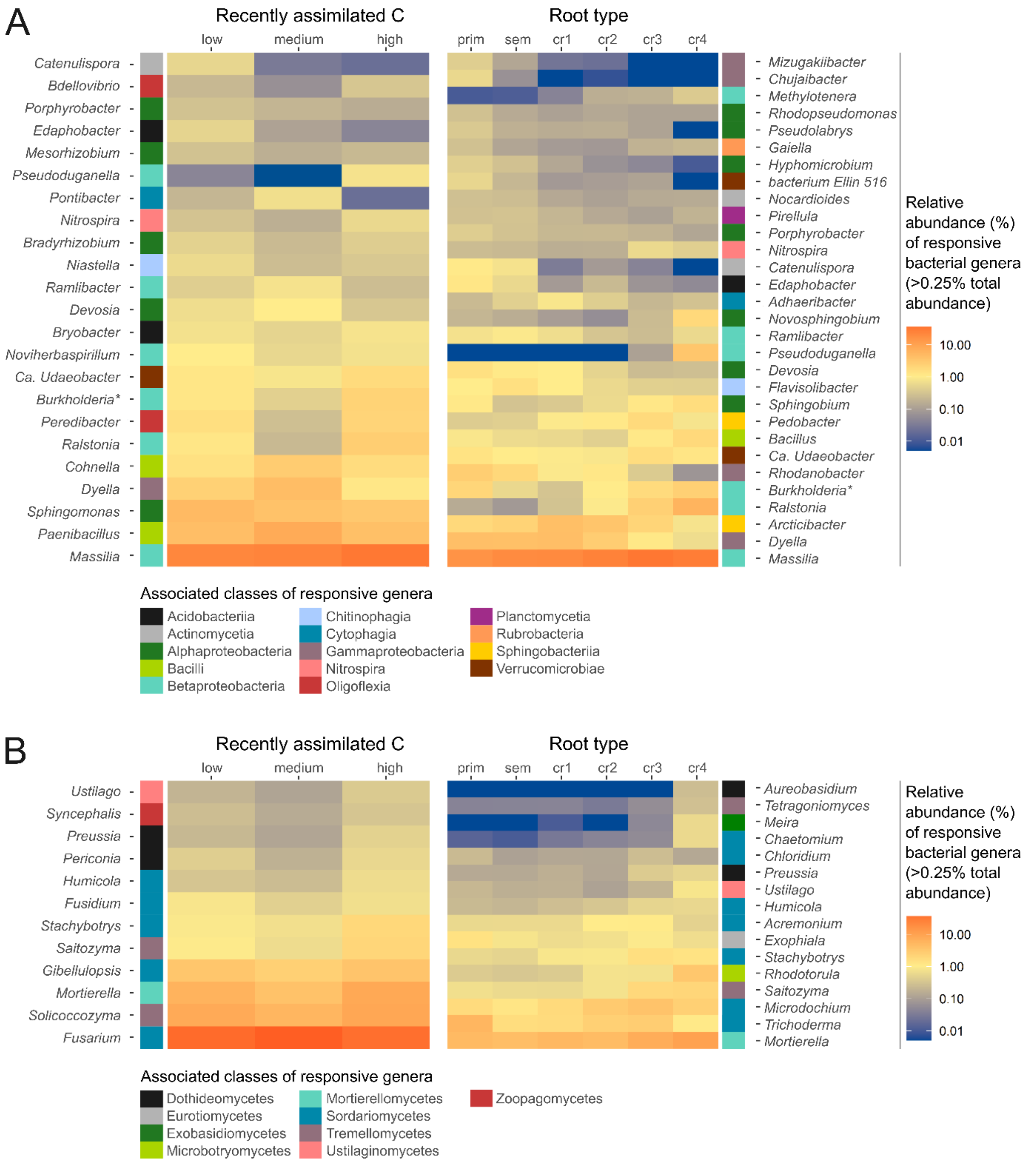

Fig. S7 Heatmaps displaying the relative abundance of dominant (>0.25% relative abundance) (**a**) bacterial and (**b**) fungal genera that showed a significant response to photosynthate level or root type. To evaluate significance, one-way ANOVA was applied with subsequent two-sided Tukey-Kramer post hoc tests and Benjamini-Hochberg correction for multiple comparisons. Detailed results of the posthoc tests are shown in Table S1 and Table S2. Bulk soil samples were excluded from this analysis. (*) Genus *Burkholderia* also encompasses members of *Paraburkholderia* and *Caballeronia*. Source data are provided as a Source Data file.

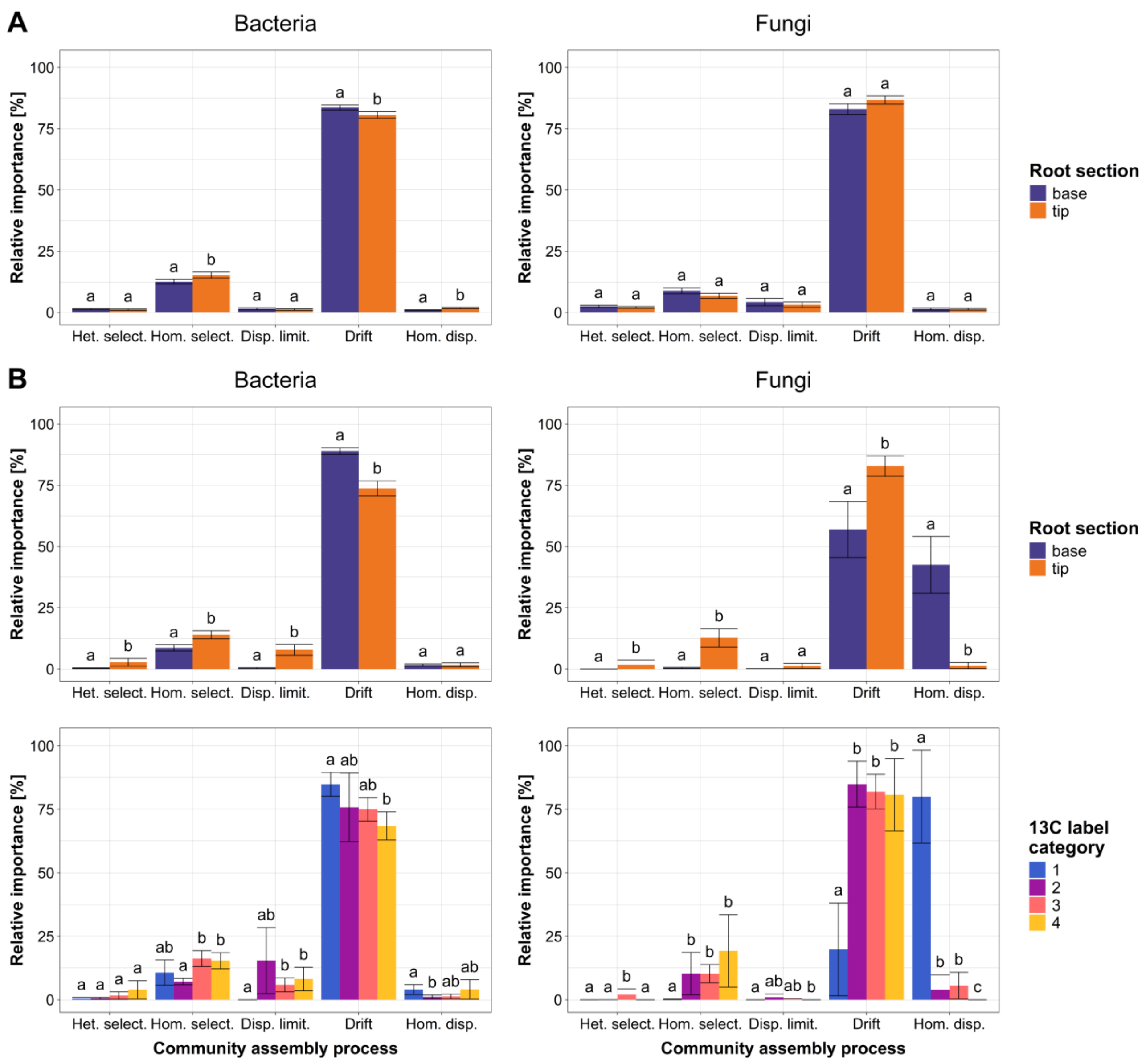

Fig. S8 Bar plots showing the relative contribution (%) of five ecological processes to bacterial and fungal community assembly. Analysis was done by iCAMP bin-based null modeling. Results are shown for **a** the total community (study I) and **b** the ^{13}C-labeled heavy fraction community (study II). Analyses were done comparatively between root sections (study I and II) and between ^{13}C label categories (study II). Each bar represents the mean ± standard deviation. Relative importance of each assembly process was statistically compared between groups based on bootstrapped results with 1000 iterations. Distinct lowercase letters indicate significant differences between groups ($p < 0.05$). Source data are provided as a Source Data file.

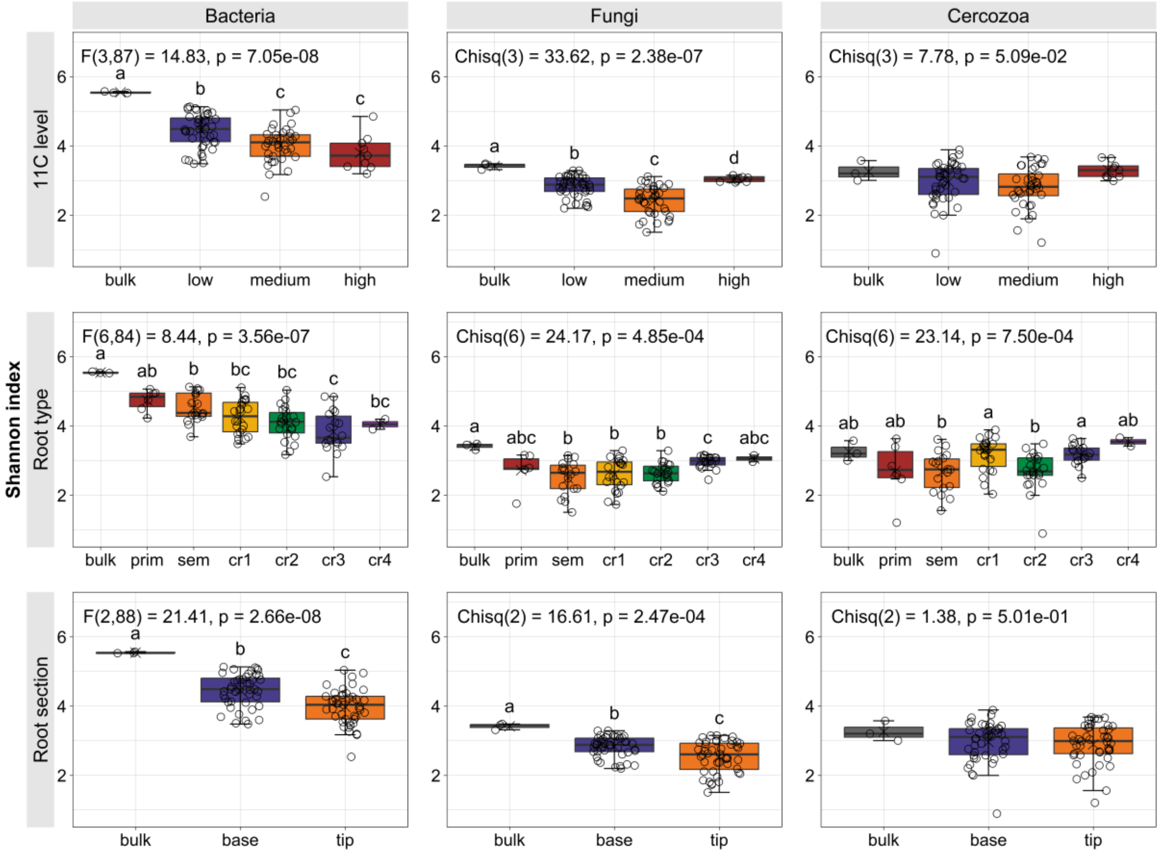

Fig. S9a The Shannon diversity index for prokaryotes, fungi and Cercozoa in dependence on root type, root section or categorical photosynthate levels according to ^{11}C-PET. Bulk = bulk soil, prim = primary root, sem = seminal roots, cr1 - cr4 = crown roots originating from nodes 1 - 4. Significance of differences was tested by one-way ANOVA and two-sided Tukey-HSD post-hoc test for the prokaryote dataset including correction for multiple comparisons. A Kruskal-Wallis test with respective two-sided Mann-Whitney U-tests including Benjamini-Hochberg correction was conducted for fungi and Cercozoa, as Shapiro-Wilk test indicated non-normal data distribution. Distinct lowercase letters indicate significant differences between samples ($p < 0.05$). Boxes span from the first to the third quartiles, the line inside each box represents the median; x represents the mean and the whiskers extend to the last data point within 1.5 times the inter quartile range. Data points outside of whiskers represent outliers. Source data are provided as a Source Data file.

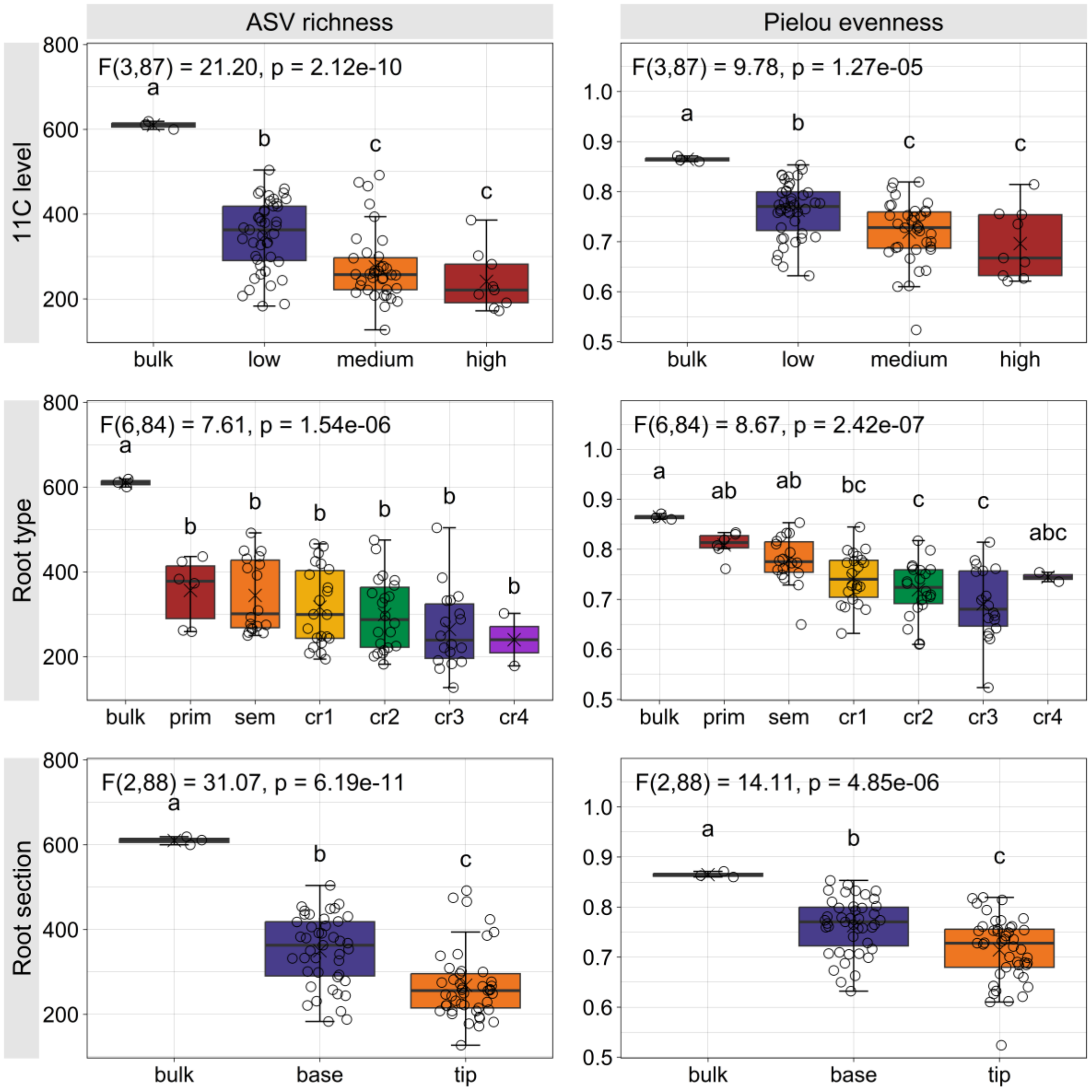

Fig. S9b The influence of photosynthate allocation, root type and root section on amplicon sequence variant richness and evenness (Pielou's index) of the prokaryotic community. Prim = primary root, sem = seminal roots, cr1 - cr4 = crown roots originating from leaf nodes 1 - 4. Significance of differences was tested by one-way ANOVA and two-sided Tukey-HSD posthoc tests with adjusted p-values controlling for multiple comparisons. Boxes span from the first to the third quartiles, the line inside each box represents the median; x represents the mean and the whiskers extend to the last data point within 1.5 times the inter quartile range. Data points outside of whiskers represent outliers. Source data are provided as a Source Data file.

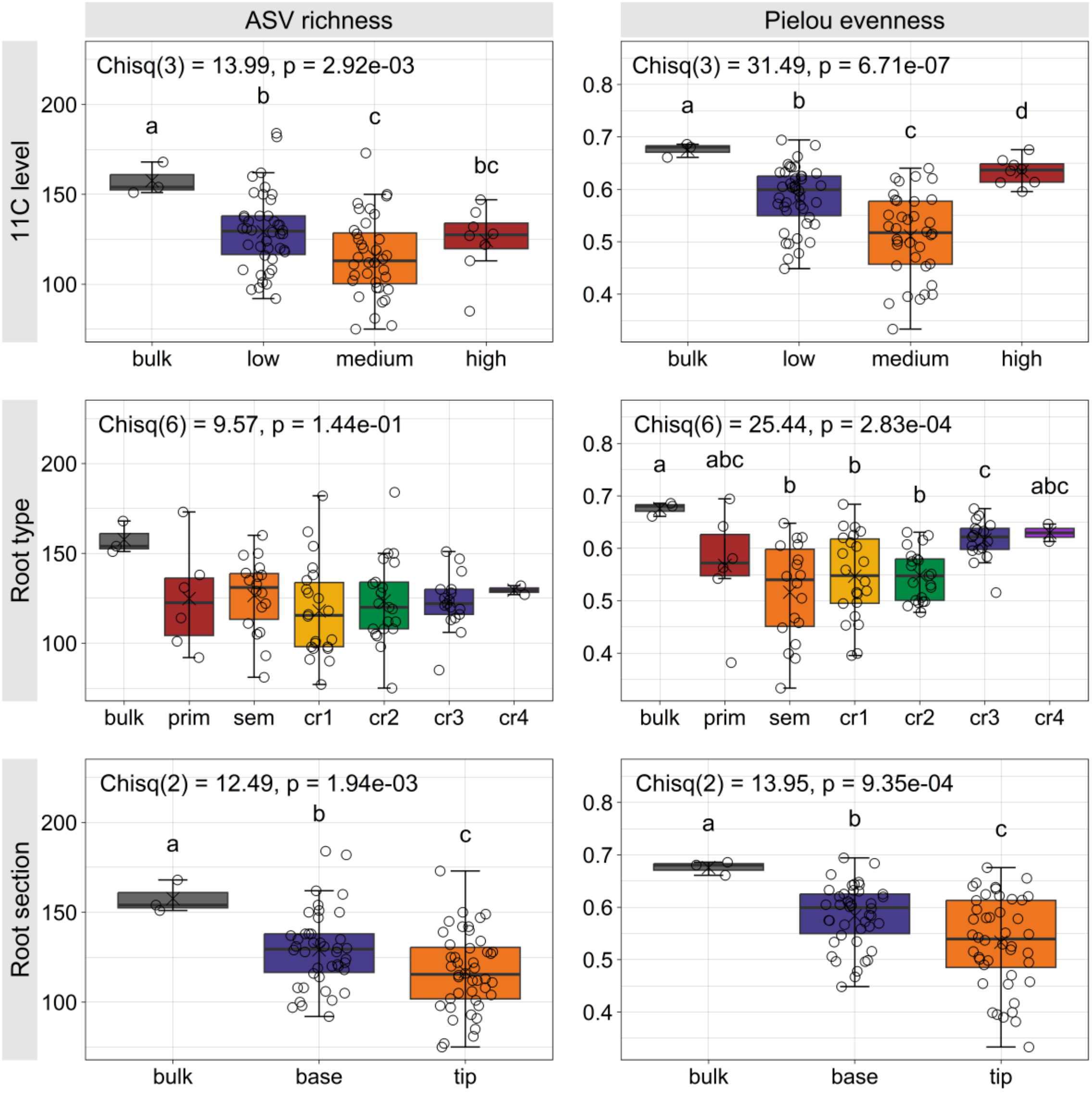

Fig. S9c The influence of photosynthate allocation, root type and root section on amplicon sequence variant richness and evenness (Pielou's index) of the fungal community. Prim = primary root, sem = seminal roots, cr1 - cr4 = crown roots originating from leaf nodes 1 - 4. Significance of differences was tested by Kruskal-Wallis test and respective two-sided Mann-Whitney U-tests with Benjamini-Hochberg correction. Boxes span from the first to the third quartiles, the line inside each box represents the median; x represents the mean and the whiskers extend to the last data point within 1.5 times the inter quartile range. Data points outside of whiskers represent outliers. Source data are provided as a Source Data file.

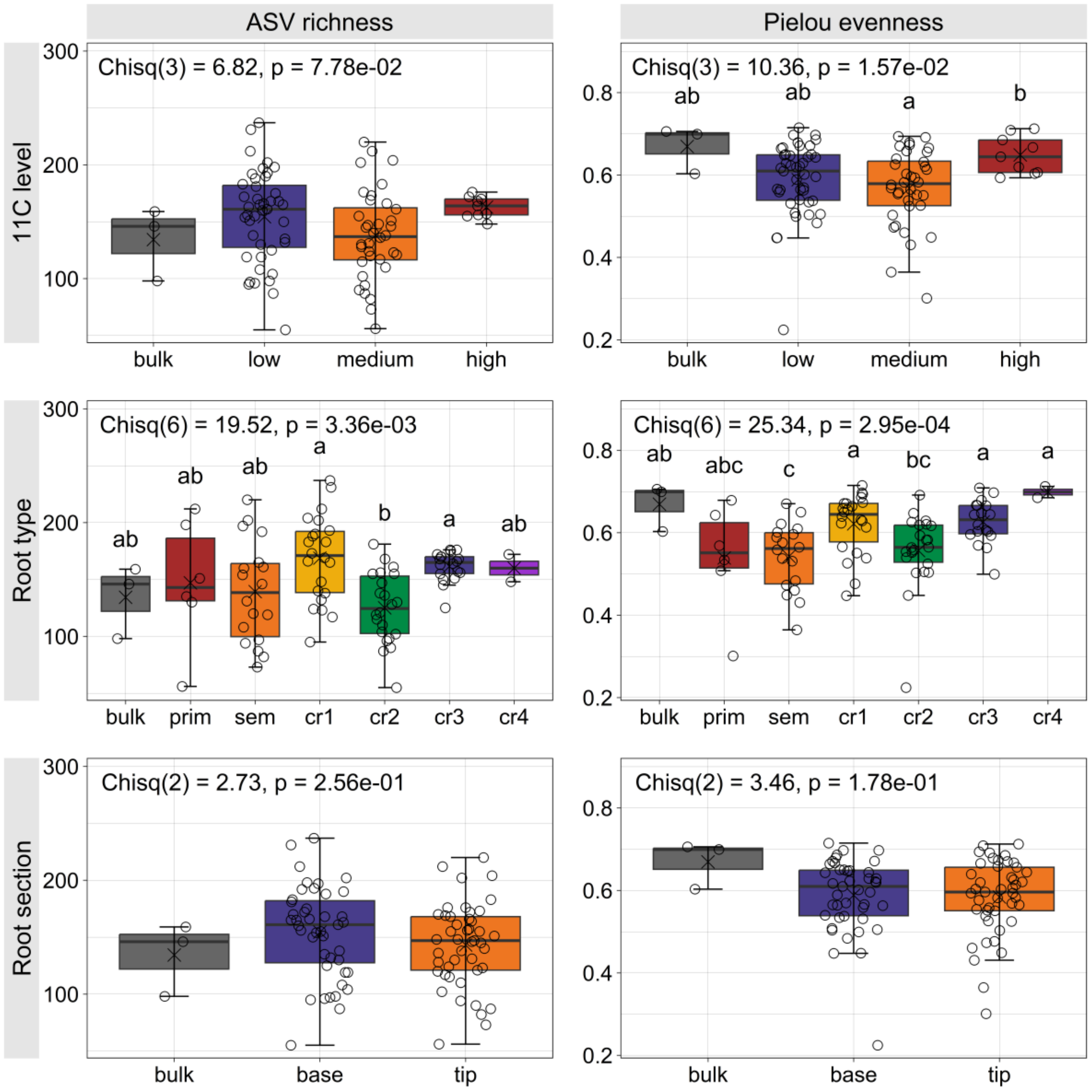

Fig. S9d The influence of photosynthate allocation, root type and root section on amplicon sequence variant richness and evenness (Pielou's index) of the Cercozoan community. Prim = primary root, sem = seminal roots, cr1 - cr4 = crown roots originating from leaf nodes 1 - 4. Significance of differences was tested by Kruskal-Wallis test and respective two-sided Mann-Whitney U-tests with Benjamini-Hochberg correction. Boxes span from the first to the third quartiles, the line inside each box represents the median; x represents the mean and the whiskers extend to the last data point within 1.5 times the inter quartile range. Data points outside of whiskers represent outliers. Source data are provided as a Source Data file.

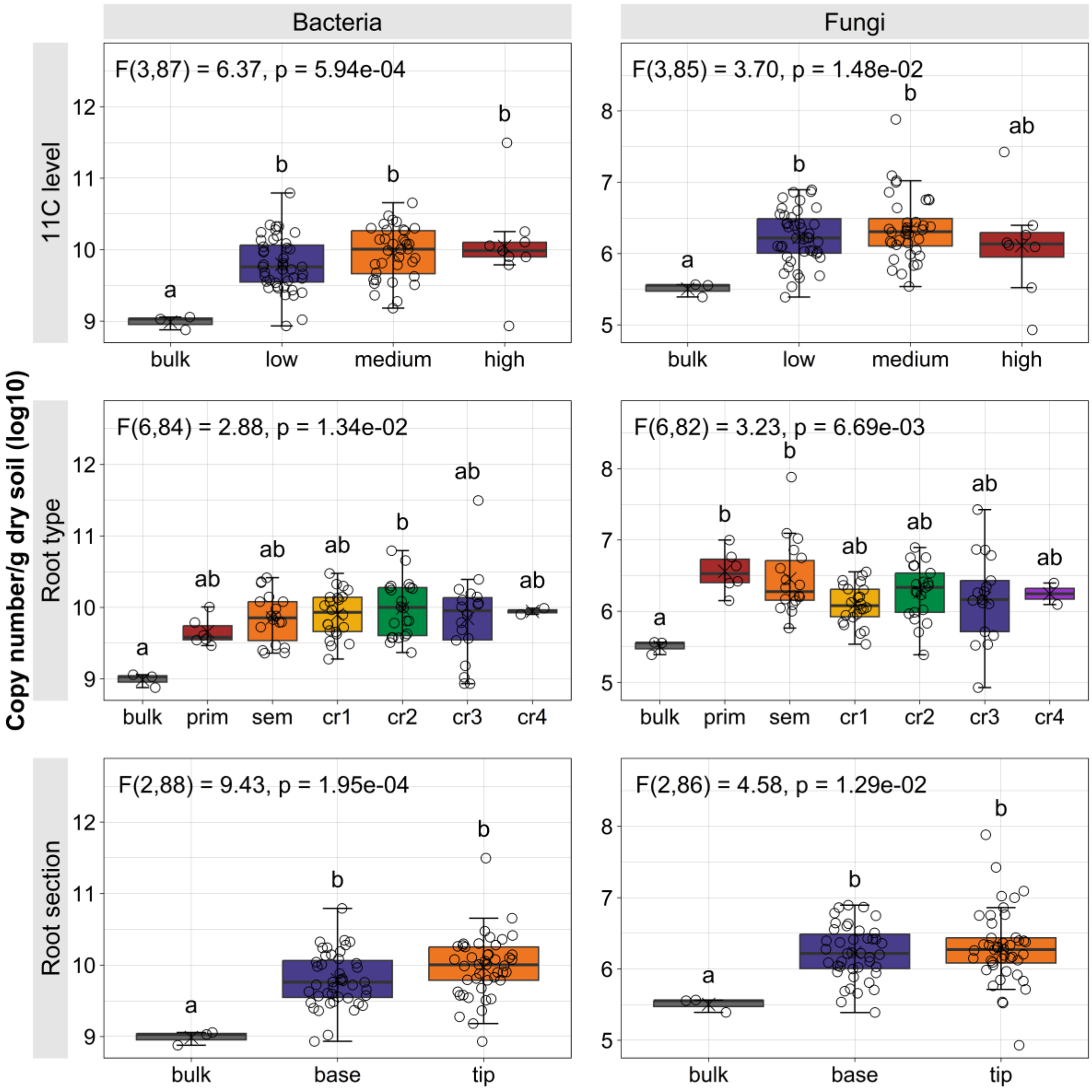

Fig. S10 Boxplots presenting the qPCR results for prokaryotic 16S rRNA gene and fungal ITS1 copy numbers and their variation in dependence on root type, root section or categorical photosynthate levels according to ^{11}C-PET. Prim = primary root, sem = seminal roots, cr1 - cr4 = crown root generations 1 - 4, originating from underground nodes 1 - 4. Significance of differences was tested by one-way ANOVA and two-sided Tukey-HSD post hoc tests with adjusted p-values controlling for multiple comparisons. Distinct lowercase letters indicate significant differences between samples ($p < 0.05$). Boxes span from the first to the third quartiles, the line inside each box represents the median; x represents the mean and the whiskers extend to the last data point within 1.5 times the inter quartile range. Data points outside of whiskers represent outliers. Source data are provided as a Source Data file.

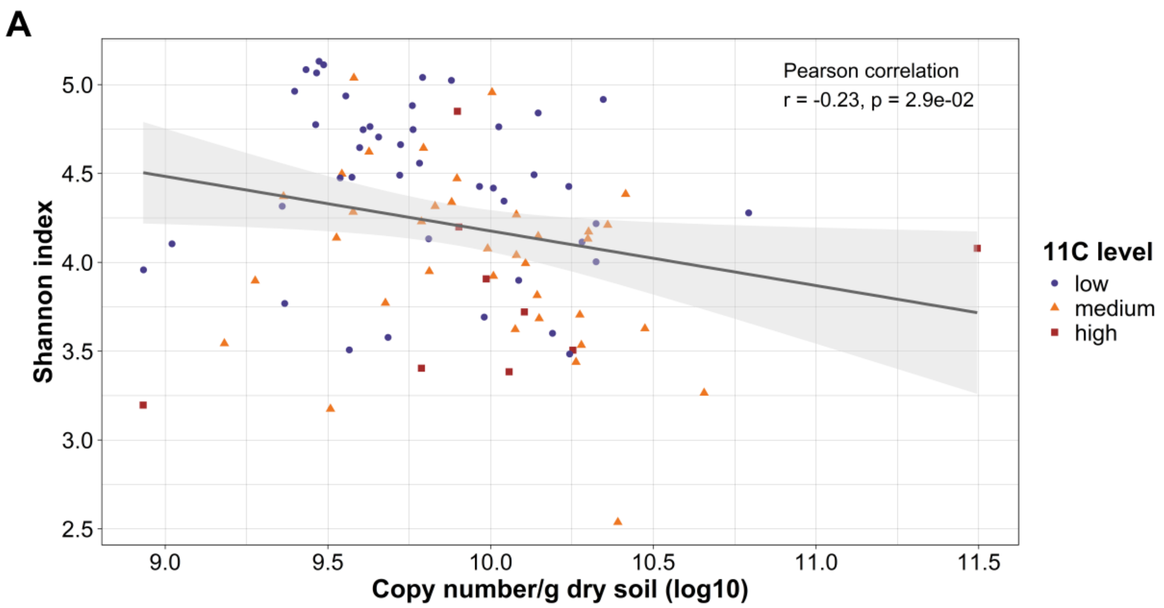

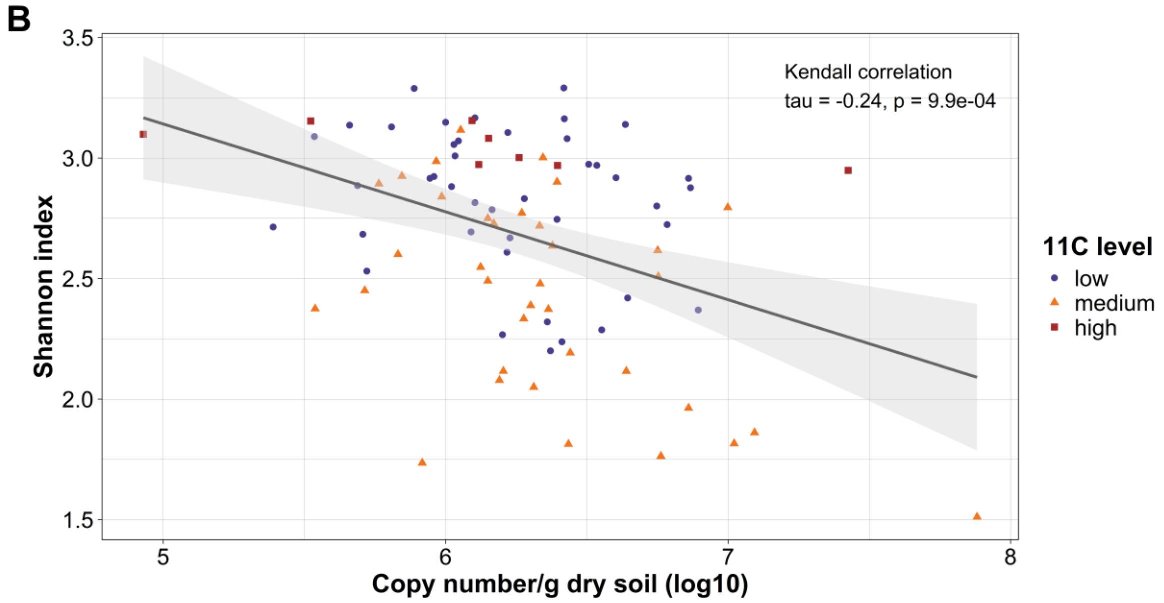

Fig. S11 Relationship between the prokaryotic (**a**) and fungal (**b**) diversity and their population size as determined by qPCR targeting a 16S rRNA gene fragment (prokaryotes) or the ITS1 region (fungi). Color coding denotes samples with different categorical photosynthate levels according to ^{11}C-PET. The number of biological replicates was *n* = 88 for prokaryotes and *n* = 86 for fungi. Gray-shaded areas indicate the 95% confidence interval of the linear regression model (solid line) fitted to the data. Correlations were verified using a Pearson correlation test for prokaryotes and a Kendall rank correlation test for the non-normally distributed fungal dataset. Source data are provided as a Source Data file.

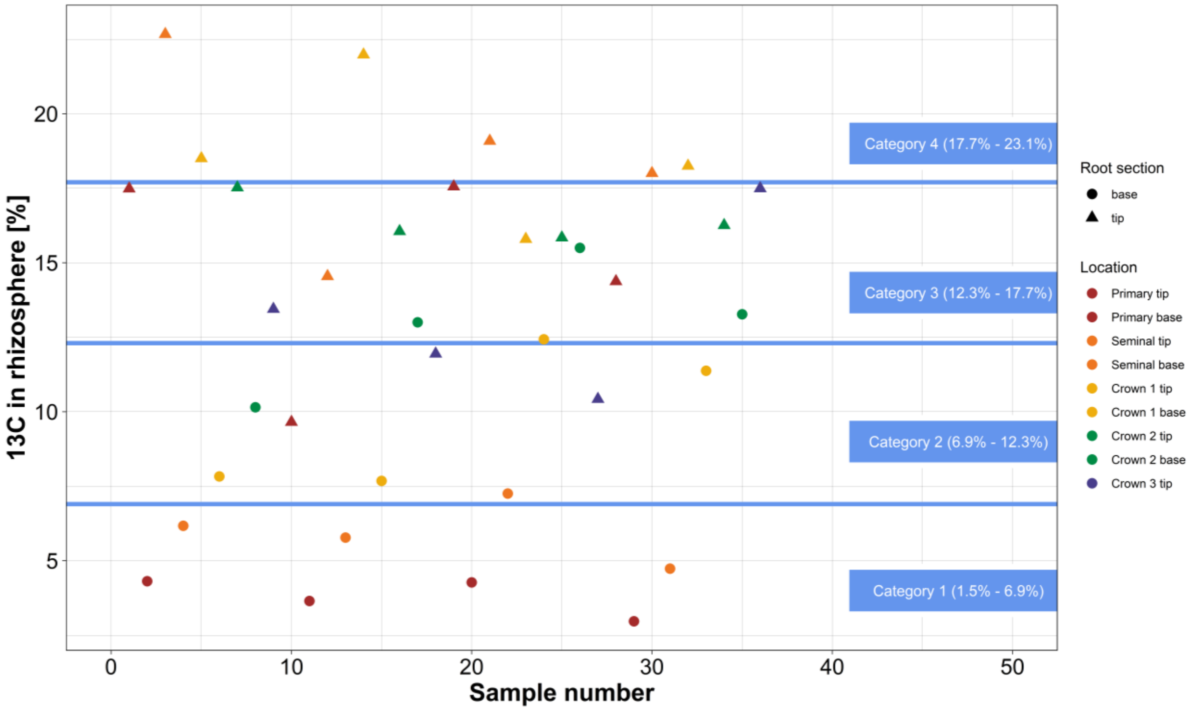

Fig. S12 Grouping of samples for HR-SIP analyses into four categories based on their ^{13}C proportion relative to total C mass in the rhizosphere soil as determined by EA-IRMS. Primary = primary root, seminal = seminal roots, Crown 1 – crown 3 = crown root generations 1 - 3, originating from underground nodes 1 – 3. Source data are provided as a Source Data file.

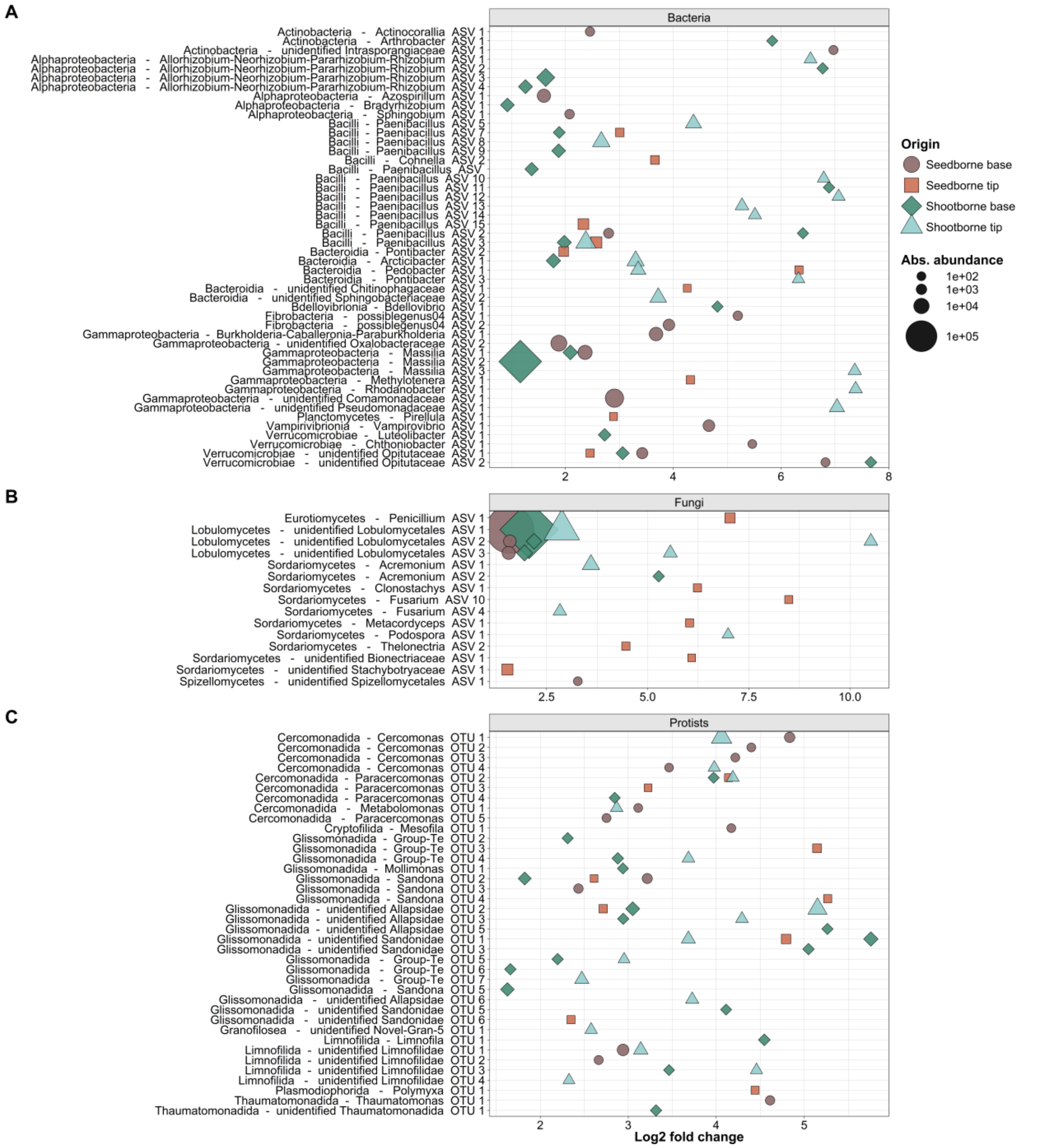

Fig. S13 ^{13}C label intensity and abundance of microbial taxa with significant label incorporation. Significantly labelled taxa were identified using the DESeq2 algorithm of the HTSSIP package. Samples were grouped into four categories in dependence on root origin in the root system (seed-borne root tips, seed-borne root bases, shoot-borne root tips, shoot-borne root bases). ^{13}C-labelling is presented as log2 fold change between the labelled-heavy and the labelled-light fraction. Absolute abundance equals the sum of all reads of a labelled ASV over all samples of a label category. Bacteria and fungi are labelled as Class_Genus_ASV and protists as Order_Genus_OTU. Source data are provided as a Source Data file.

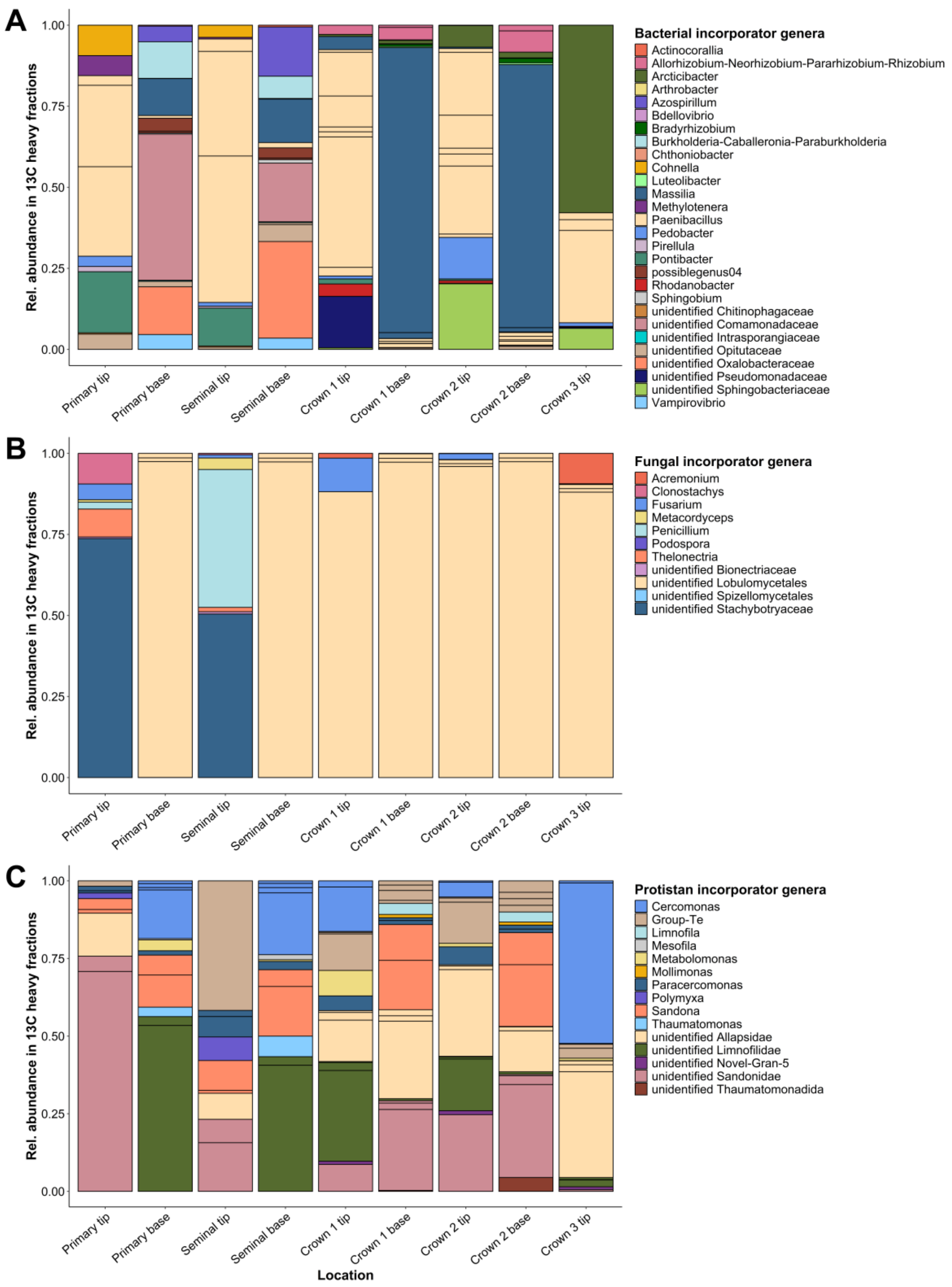

Fig. S14 Relative abundance of ^{13}C-labeled taxa at different locations in the root system. Taxa with significant ^{13}C label incorporation were identified by HR-SIP with replicate samples grouped based on their origin in the root system (seed-borne root tip, seed-borne root base, shoot-borne root tip, shoot-borne root base). Relative abundance was calculated based on sequence read counts in the ^{13}C-heavy fractions of replicate samples per root type/section. Different ASVs within the same genus are shown in the same color. Source data are provided as a Source Data file.

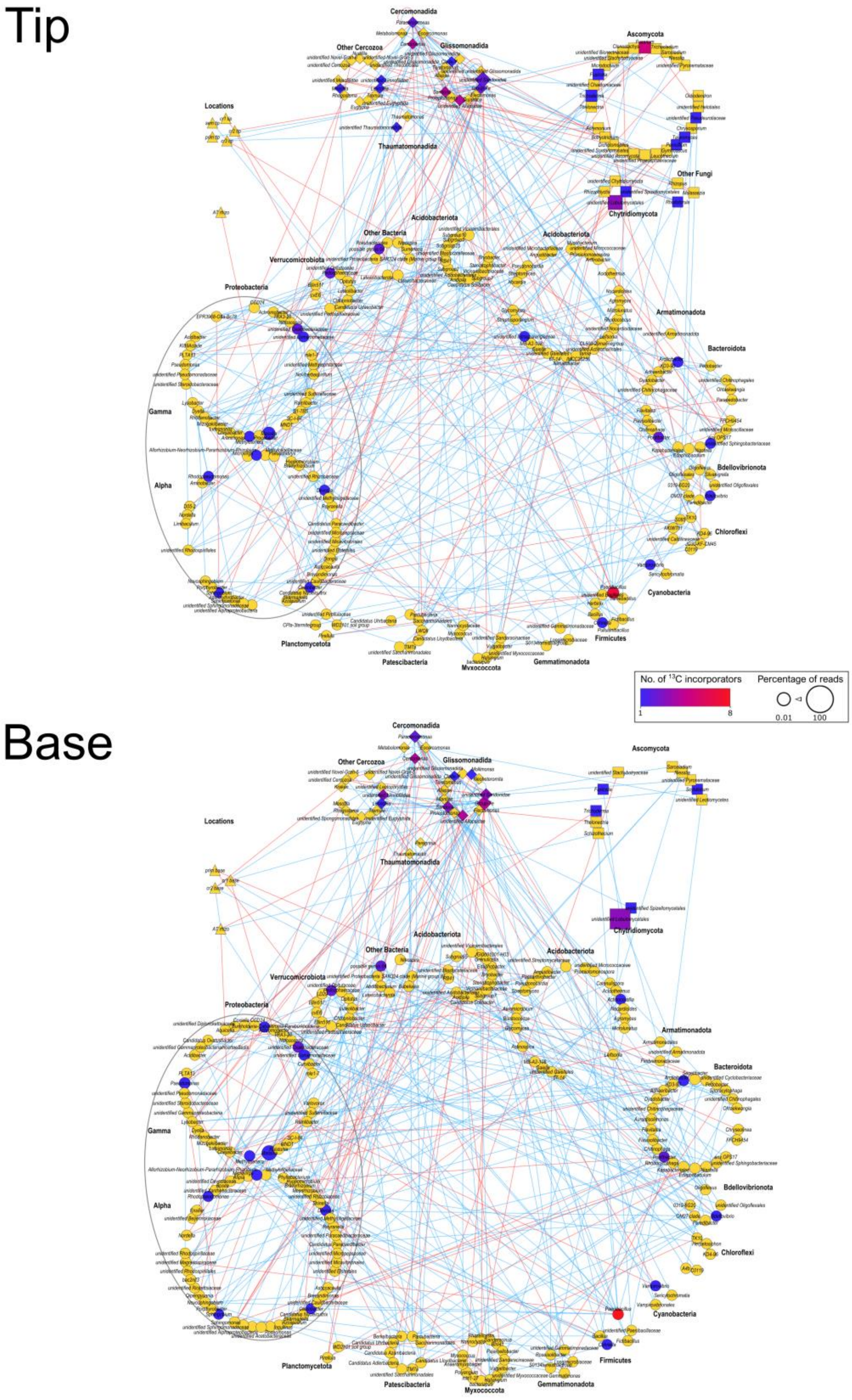

Fig. S15 Microbial co-occurrence networks resolved at genus level. The same networks for root tip and root base are shown here as in Fig. 6, but at higher taxonomic resolution. Positive associations are indicated as blue lines, negative associations as red lines. ^{13}C-incorporators are highlighted in a color range from blue to red depending on the number of labeled ASVs in the genus, non-incorporators are shown in yellow. The node size is proportional to the percentage of reads. Source data are provided as a Source Data file.

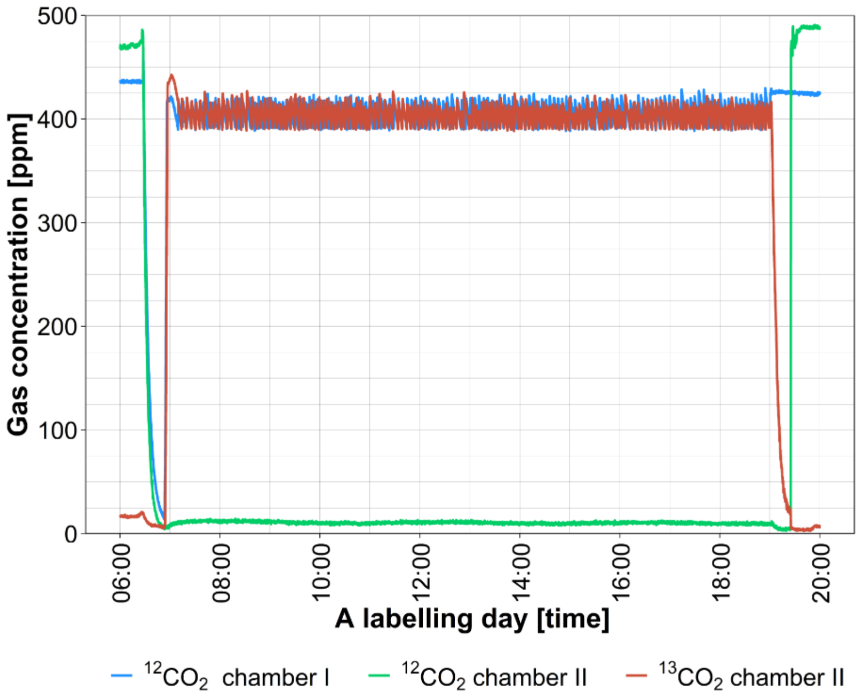

Fig. S16 Automatically regulated gas flow during a labelling day in the $^{13}CO_2$ labelling chamber (chamber II) and the $^{12}CO_2$ control chamber (chamber I). Gas concentrations were maintained at a constant level using a customized automatic valve regulation system, controlled based on measurements with the extractive gas analyzers LI-820 (LI-COR Biosciences, Lincoln, NE, USA) and S710 (SICK AG, Waldkirch, Germany). Gas exchange data were recorded in 10 second intervals. Temperature in both chambers was regulated using a DKRF400 sensor (Driesen+Kern GmbH, Bad Bramstedt, Germany) and a Julabo FN25-HE water cooling unit (Julabo GmbH, Seelbach, Germany). Source data are provided as a Source Data file.

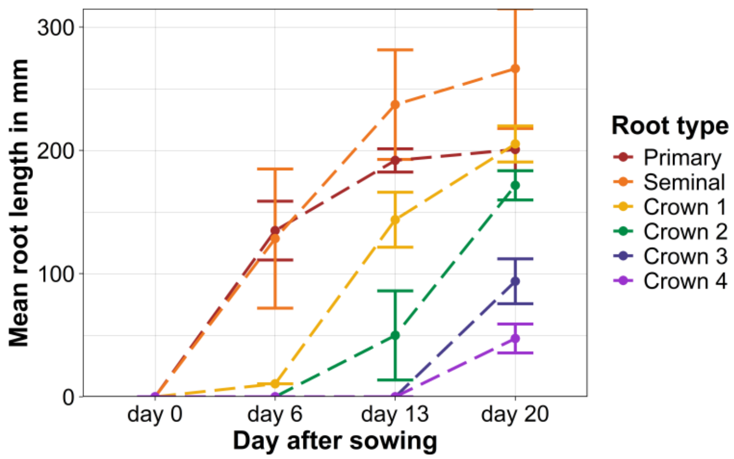

Fig. S17 Mean root length (mm) of the examined primary, seminal and crown root types from the four different leaf nodes, as determined by MRI measurements and NMRooting analysis at day 6, 13 and 20 after sowing (study I). Error bars denote the standard deviation. Source data are provided as a Source Data file.

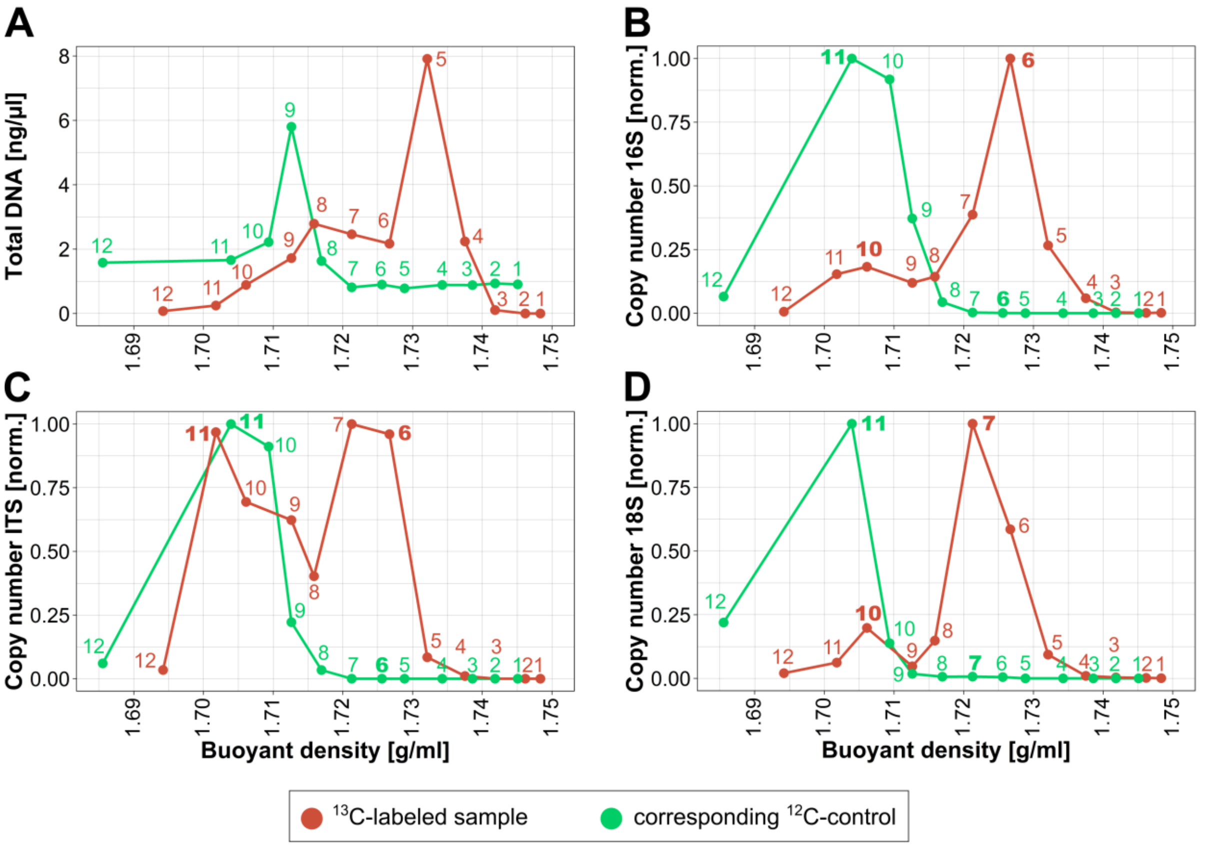

Fig. S18 A ^{13}C-labeled sample and its corresponding ^{12}C-control sample from the crown root 1 tip as an example for SIP fraction selection prior to amplicon sequencing. (**a**) The total DNA content and buoyant density of all fractions (numbered). (**b-d**) 16S rRNA gene, ITS1 region and 18S rRNA gen copy numbers of all fractions as determined by qPCR, normalized to 1.00. Fractions for amplicon sequencing were selected based on the qPCR results and corresponding numbers are printed in bold. Source data are provided as a Source Data file.

Supplementary tables

Table S1 Detailed test statistics accompanying the PERMANOVA results in Table 1. Df = degrees of freedom, SumOfSqs = sum of squares, R² = effect size, F = F-statistic from the permutation test, Pr(>F) = permutation-based *p*-value based on 999 permutations.

Study	Organism	Model	Term	Df	SumOfSqs	R2	F	Pr(>F)
Study 1	Bacteria	Root type * ^{11}C level	Root type	5	3.056365	0.198823	4.534046	0.001
			^{11}C level	2	1.058651	0.068867	3.926212	0.001
			Interaction	4	1.011086	0.065773	1.874904	0.001
			Residuals	76	10.2462	0.666536		
			Total	87	15.3723	1		
	Fungi	Root type * ^{11}C level	Root type	5	1.156018	0.133403	2.809233	0.001
			^{11}C level	2	0.734551	0.084766	4.462569	0.001
			Interaction	4	0.684737	0.079018	2.07997	0.007
			Residuals	74	6.0903	0.702813		
			Total	85	8.665606	1		
	Cercozoa	Root type * ^{11}C level	Root type	5	2.600377	0.177276	3.913342	0.001
			^{11}C level	2	1.387028	0.094558	5.218393	0.001
			Interaction	4	0.580892	0.039601	1.09274	0.325
			Residuals	76	10.10025	0.688565		
			Total	87	14.66854	1		
Study 2	Bacteria	Root type * ^{11}C level	Root type	4	1.727565	0.255125	6.228258	0.001
			^{11}C level	1	2.332357	0.344440	33.63467	0.001
			Interaction	3	0.908582	0.134179	4.367523	0.001
			Residuals	26	1.80294	0.266256		
			Total	34	6.771445	1		
		Root type * ^{13}C level	Root type	4	1.727565	0.255125	4.967874	0.001
			^{13}C level	1	2.105692	0.310966	24.22094	0.001
			Interaction	4	0.764767	0.11294	2.199202	0.008
			Residuals	25	2.173421	0.320969		
			Total	34	6.771445	1		
	Fungi	Root type * ^{11}C level	Root type	4	0.911765	0.141359	2.136404	0.026
			^{11}C level	1	2.278835	0.353309	21.35863	0.001
			Interaction	3	0.912118	0.141414	2.849642	0.007
			Residuals	22	2.347266	0.363918		
			Total	30	6.449985	1		
		Root type * ^{13}C level	Root type	4	0.911765	0.141359	1.777263	0.052
			^{13}C level	1	2.198003	0.340776	17.13788	0.001
			Interaction	4	0.646882	0.100292	1.260938	0.26
			Residuals	21	2.693335	0.417572		
			Total	30	6.449985	1		
	Cercozoa	Root type * ^{11}C level	Root type	4	1.078341	0.167676	3.631746	0.005
			^{11}C level	1	3.021937	0.469894	40.71034	0.001
			Interaction	3	0.326604	0.050785	1.466628	0.177
			Residuals	27	2.004215	0.311644		
			Total	35	6.431098	1		
		Root type * ^{13}C level	Root type	4	1.078341	0.167676	2.689224	0.015
			^{13}C level	1	2.407528	0.374357	24.01608	0.001
			Interaction	4	0.33882	0.052685	0.844968	0.57
			Residuals	26	2.606409	0.405282		
			Total	35	6.431098	1		

Table S2 The effect of root type and photosynthate allocation on responsive fungal genera in study I as identified by one-way ANOVA (two-sided) with Benjamini-Hochberg correction. Shown are the detailed results of Tukey-Kramer posthoc tests. Different letters indicate significant differences between groups ($p < 0.05$). Genera printed in bold responded to both root type and photosynthate allocation. Grey shaded fields indicate that a genus did not respond to a factor. Only genera > 0.25% relative abundance are included in Fig. S6.

Fungal genus	Root type						Photosynthate level		
	prim	sem	cr1	cr2	cr3	cr4	low	medium	high
Acremonium	a	a	a	a	a	b			
Actinomucor	a	a	a	a	a	b			
Aureobasidium	a	a	a	a	a	b	a	a	b
Buckleyzyma							a	a	b
Chaetomium	a	a	a	a	a	b	a	a	b
Chloridium	ab	a	a	a	b	ab	a	b	ab
Chrysosporium	a	a	ab	ab	b	ab			
Clavulina	a	a	a	a	a	b	a	a	b
Exophiala	a	b	b	b	ab	ab			
Fusarium							a	b	a
Fusidium							a	b	ab
Gibellulopsis							a	b	a
Humicola	ab	a	a	ab	b	ab	a	a	b
Hypholoma	a	a	a	a	a	b			
Leptobacillium	a	b	b	b	b	ab			
Lobulomyces	a	a	a	a	a	b			
Lophotrichus							a	a	b
Lycoperdon	a	a	a	a	a	b			
Meira	a	a	a	a	a	b			
Microdochium	ab	a	a	bc	c	abc			
Mortierella	abc	a	abc	a	b	bc	a	b	c
Periconia							a	b	a
Phoma	ab	a	a	ab	b	c			
Preussia	a	a	a	a	b	bc	a	b	c
Psathyrella	a	a	a	a	b	ab	a	a	b
Rhodotorula	a	a	a	a	a	b			
Roussoella	ab	ab	a	a	b	ab			
Saitozyma	a	a	a	a	b	bc	a	a	b
Sclerotinia	a	a	a	a	a	b			
Solicoccozyma							a	b	a
Spizellomyces	a	ab	a	a	a	b			
Stachybotrys	a	a	a	a	b	ab	a	a	b
Sterigmatomyces							a	a	b
Syncephalis							a	b	a
Tetracladium							a	b	a
Tetragoniomyces	a	a	a	a	a	b			
Thielavia	a	a	a	a	a	b			
Tomentella	a	a	a	a	a	b	a	a	b
Trichoderma	a	b	b	b	ab	ab			
Ustilago	a	a	a	a	a	b	a	a	b
Verticillium	a	a	a	a	a	b			

Table S3 Influence of photosynthate level and root type on microbial community composition of DNA fractions collected during DNA-SIP in study II. The influence of both factors as well as the interaction on community composition was assessed by PERMANOVA based on 999 permutations (R^2 values given in table). Factor root section was consistently eliminated in the PERMANOVA models. Significance code '***' indicates *p* values ≤0.001, ** *p* values ≤0.01 and * *p* values ≤0.05.

		**Model: Root type * ^{11}C level**				**Model: Root type * ^{13}C level**			
**Group**	**DNA Fraction**	**^{11}C-level (cat.)**	**Root type**	**Inter-action**	**Residuals**	**^{13}C level (num.)**	**Root type**	**Inter-action**	**Residuals**
Bacteria Archaea	^{13}C light	0.28***	0.27***	0.09*	0.36	0.24***	0.27***	0.08	0.41
	^{12}C heavy	0.22***	0.36***	0.13*	0.29	NA	NA	NA	NA
	^{12}C light	0.20***	0.29***	0.10**	0.41	NA	NA	NA	NA
Fungi	^{13}C light	0.34***	0.12	0.10*	0.44	0.29***	0.12	0.17***	0.42
	^{12}C heavy	0.26***	0.22**	0.10	0.42	NA	NA	NA	NA
	^{12}C light	0.14*	0.32***	0.09	0.45	NA	NA	NA	NA
Cercozoa	^{13}C light	0.39***	0.17**	0.08*	0.36	0.32***	0.17**	0.09	0.42
	^{12}C heavy	0.25***	0.17**	0.09	0.49	NA	NA	NA	NA
	^{12}C light	0.41***	0.15**	0.06	0.38	NA	NA	NA	NA

Table S4 Number of different ASVs/OTUs detected as “labelled” by DESeq2 analysis within each taxon and per category. Categories were defined based on mass fraction of ^{13}C in the rhizosphere soil or origin in root system as grouping factor.

Grouping factor	Category	# bacterial ASVs	# fungal ASVs	# cercozoan OTUs
^{13}C level	Category 1 (1.5 – 6.9%)	14	4	10
	Category 2 (6.9 – 12.3%)	10	2	11
	Category 3 (12.3 – 17.7%)	7	6	9
	Category 4 (17.7 – 23.1%)	12	14	12
Origin	Seedborne root tip	10	7	9
	Seedborne root base	15	4	12
	Shootborne root tip	15	6	15
	Shootborne root base	18	4	18

Table S5 Comparison of microbial co-occurrence network parameters between ^{13}C incorporators and non-incorporators. Significance of differences between groups was tested by two-sided Wilcoxon rank sum test.

Parameter	Base					Tip				
	Incorporators		No Incorporators		Wilcoxon Rank *p* value	Incorporators		No Incorporators		Wilcoxon Rank *p* value
	Mean	SD	Mean	SD		Mean	SD	Mean	SD	
Eccentricity	14.2	4.6	15.9	4.4	**<0.001**	15.2	6.5	17.0	5.2	0.109
Degree	2.6	1.3	2.2	1.3	**0.011**	2.1	1.1	2.2	1.3	0.890
Radiality	0.32	0.22	0.23	0.25	**0.013**	0.01	0.45	-0.10	0.38	0.059
Average Shortest Path Length	7.1	2.1	7.9	2.3	**0.013**	7.8	3.4	8.6	2.8	0.059
Closeness Centrality	0.20	0.22	0.17	0.20	**0.013**	0.23	0.27	0.17	0.21	0.059
Betweenness Centrality	0.06	0.19	0.02	0.06	**0.013**	0.06	0.17	0.03	0.10	0.905
Stress	4770	5850	3330	4705	**0.029**	3294	4416	3893	5720	0.604

Table S6 Number of roots per root type of each plant on days 14 and 21 after sowing as determined by MRI in study II. Plant pairs xA and xB were pooled for sampling on day 22.

Plant ID	^{13}C-labeled/ ^{12}C-control	Day after sowing	# of seminal roots	# of crown 1 roots	# of crown 2 roots	# of crown 3 roots
1A	^{13}C-labeled	14	3	3	3	0
		21	3	3	3	4
1B	^{13}C-labeled	14	3	3	3	0
		21	3	3	3	4
2A	^{13}C-labeled	14	4	4	2	0
		21	4	4	3	4
2B	^{13}C-labeled	14	3	4	1	0
		21	3	4	3	3
3A	^{13}C-labeled	14	3	4	0	0
		21	3	4	3	3
3B	^{13}C-labeled	14	3	5	0	0
		21	3	6	3	2
4A	^{13}C-labeled	14	3	3	2	0
		21	3	3	2	3
4B	^{13}C-labeled	14	3	3	0	0
		21	3	3	3	4
5A	^{12}C-control	14	3	3	3	0
		21	3	3	3	3
5B	^{12}C-control	14	3	4	2	0
		21	3	4	4	4
6A	^{12}C-control	14	3	4	0	0
		21	3	4	4	3
6B	^{12}C-control	14	4	3	2	0
		21	4	3	3	4
7A	^{12}C-control	14	3	3	0	0
		21	3	3	3	3
7B	^{12}C-control	14	3	3	2	0
		21	3	3	3	4
8A	^{12}C-control	14	3	4	0	0
		21	3	4	4	3
8B	^{12}C-control	14	3	3	1	0
		21	3	4	4	3

Table S7 Primers used to generate standards and cycling conditions for qPCR. Primer sequences available via reference list for bacteria/archaea[1], fungi[2] and Cercozoa[3].

		**Bacteria/archaea**		**Fungi**		**Cercozoa**	
Standard	Target region	16S rRNA		ITS1		18S rRNA (V4)	
	Primers	9f/1492r		ITS1f/ITS4		S615F_Cer/S947R_Cer	
Cycling	Initial denaturation	2:00 min, 95°C		2:00 min, 95°C		2:00 min at 95°C	
	Cycles including quantification steps	40x	0:10 min, 95°C<br>0:45 min, 60°C	40x	0:10 min, 95°C<br>0:45 min, 58°C	40x	0:10 min, 95°C<br>0:45 min, 57°C
	Optional read step		0:05 min, 84°C		-		0:05 min, 83.5°C

Table S8 Primers for amplicon sequencing and qPCR of prokaryotes (bacteria and archaea)[4], fungi[5, 6] and protists (Cercozoa)[3]. BC indicates barcoded primers.

**Organisms**	**Target region**	**Primer sets round 1**	**Primer sets round 2**
Bacteria/ archaea	V4-V5 region of 16S SSU rRNA gene	**515f:**<br>5'-GTGCCAGCMGCCGCGGTAA-3'<br>**806fr**<br>5'-GGACTACHVGGGTWTCTAAT-3'	**515f (BC)**<br>**806r**
Fungi	ITS1 region of nuclear DNA	**ITS1F:**<br>5'-CTTGGTCATTTAGAGGAAGTAA-3'<br>**ITS2:**<br>5'-GCTGCGTTCTTCATCGATGC-3'	**ITS1F (BC)**<br>**ITS2**
Cercozoa	V4 region of 18S SSU rRNA gene	**S615F_Cerco:**<br>5'-GTTAAAAAGCTCGTAGTTG-3'<br>**S615F_Phyt:**<br>5'-GTTAAAARGCTCGTAGTCG -3'<br>**S963R_Phyt:**<br>5'-CAACTTTCGTTCTTGATTAAA-3'	**S615F_Cer (BC):**<br>5' GTTAAAARGCTCGTAGTYG-3'<br>**S947R_Cer (BC):**<br>5'-AAGARGAYATCCTTGGTG-3'

Table S9 Two-step PCR conditions for amplicon sequencing of prokaryotes, fungi and Cercozoa.

	Bacteria/archaea		Fungi		Cercozoa	
**Round 1**						
Initial denaturation	2:00 min, 95°C		1:00 min, 95°C		2:00 min, 95°C	
Denaturation		0:20 min, 95°C		0:20 min, 95°C		0:30 min, 95°C
Annealing	25x	0:20 min, 52°C	30x	0:20 min, 55°C	24x	0:30 min, 50°C
Elongation		0:20 min, 72°C		0:20 min, 72°C		0:30 min, 72°C
Final elongation	3:00 min, 72°C		3:00 min, 72°C		5:00 min, 72°C	
**Round 2**						
Initial denaturation	2:00 min, 95°C		1:00 min, 95°C		2:00 min, 95°C	
Denaturation		0:20 min, 95°C		0:20 min, 95°C		0:30 min, 95°C
Annealing	6x	0:20 min, 52°C	6x	0:20 min, 55°C	24x	0:30 min, 50°C
Elongation		0:20 min, 72°C		0:20 min, 72°C		0:30 min, 72°C
Final elongation	3:00 min, 72°C		3:00 min, 72°C		5:00 min, 72°C	

Table S10 PCR reaction composition for amplicon sequencing.

Chemical	PCR Round 1 (10 µl)	PCR Round 2 (30 µl)
5 x Herculase II Fusion buffer	1x	1x
dNTPs	0.25 mM each	0.25 mM each
primer A (barcoded in round 2)	0.25 µM	0.25 µM
primer B	0.25 µM	0.25 µM
$MgCl_2$	1 mM	1 mM
BSA	0.8 mg/ml	0.8 mg/ml
Herculase II Fusion Polymerase	0.5 U	1.5 U
DNA template	1 µl	1 µl round 1 product (16S) or 2 µl purified product (ITS)

Table S11 Total and mean read number per sample remaining for downstream analyses after initial quality filtering.

Target gene	Study I		Study II	
	Total reads	Mean / sample	Total reads	Mean / sample
16S rRNA gene	1702486	18708	3987467	31152
ITS1 region	3762210	42272	3119812	24958
18S rRNA gene	4794021	52681	3145803	21845

Supplementary references

1. Heyer J, Galchenko VF, Dunfield PF. Molecular phylogeny of type II methane-oxidizing bacteria isolated from various environments. *Microbiology* **148**, 2831-2846 (2002).

2. Manter DK, Vivanco JM. Use of the ITS primers, ITS1F and ITS4, to characterize fungal abundance and diversity in mixed-template samples by qPCR and length heterogeneity analysis. *Journal of Microbiological Methods* **71**, 7-14 (2007).

3. Fiore-Donno AM, Richter-Heitmann T, Bonkowski M. Contrasting responses of protistan plant parasites and phagotrophs to ecosystems, land management and soil properties. *Frontiers in Microbiology* **11**, 1823 (2020).

4. Caporaso JG*, et al.* Global patterns of 16S rRNA diversity at a depth of millions of sequences per sample. *PNAS* **108**, 4516-4522 (2011).

5. White TJ, Bruns T, Lee S, Taylor J. Amplification and direct sequencing of fungal ribosomal RNA genes for phylogenetics. In: *PCR Protocols: A Guide to Methods and Applications* (eds Innis MA, Gelfand DH, Sninsky JJ, TJ W). Academic Press, London (1990).

6. Gardes M, Bruns TD. ITS primers with enhanced specificity for basidiomycetes-application to the identification of mycorrhizae and rusts. *Molecular Ecology* **2**, 113-118 (1993).
