# Supplementary material for: Photosynthate distribution determines spatial patterns in the rhizosphere microbiota of the maize root system
Source: Nat Commun. 2025 Aug 7;16:7286. doi: 10.1038/s41467-025-62550-y (PMC12331955; doi:10.1038/s41467-025-62550-y)
Supplement: Supplementary file 2 — Description of Additional Supplementary Files [file 41467_2025_62550_MOESM2_ESM.pdf]

Description of Additional Supplementary Files

Supplementary Data 1: The effect of root type and photosynthate allocation on responsive prokaryotic genera of study I as identified by one-way ANOVA (two-sided) with BenjaminiHochberg correction. Shown are the detailed results of Tukey-Kramer posthoc tests. Different letters indicate significant differences between groups ($p < 0.05$). Genera printed in bold responded to both, root type and photosynthate allocation. Grey shaded fields indicate that a genus did not respond to a factor. Only genera > 0.25% relative abundance are included in Fig. S6.

Supplementary Movie 1: Allocation of recently fixed carbon in maize roots. Allocation of 11Clabeled carbon into the maize root system as visualized by PET/MRI imaging for an individual plant in study I. The same maize plant was imaged with an age of 6, 13, and 20 days. Coregistered PET-MRI scans show 11C allocation according to PET (colored) within the root system as seen by MRI (grey) over a period of 120 minutes upon label application.
